# Effects of *Lactiplantibacillus plantarum* and *Lactiplantibacillus brevis* on fermentation, aerobic stability, and the bacterial community of paper mulberry silage

**DOI:** 10.3389/fmicb.2022.1063914

**Published:** 2022-11-22

**Authors:** Yulin Zhang, Hanjun Yang, Rongzheng Huang, Xuzhe Wang, Chunhui Ma, Fanfan Zhang

**Affiliations:** ^1^Grass Land Science, College of Animal Science and Technology, Shihezi University, Shihezi, China; ^2^Grass Land Science, College of Animal Science and Technology, Tarim University, Alar, China

**Keywords:** lactic acid bacteria, paper mulberry, fermentation, bacterial community, aerobic stability

## Abstract

The present study investigated the dynamic profiles of fermentation quality, aerobic stability, and the bacterial community of paper mulberry silage inoculants without (Control) or with *Lactiplantibacillus plantarum* (LP), *Lactiplantibacillus brevis* (LB), or their combination (LPLB), which was screened from naturally fermented paper mulberry. The results showed that the inoculated treatments had significantly reduced neutral detergent fiber, water-soluble carbohydrates, and ammoniacal nitrogen contents compared with the control after 60 days of ensiling (the decreased proportion of LP, LB, and LPLB treatments ranged from 7.33%–11.23%, 9.60%–21.44%, and 21.53%–29.23%, respectively, *p <* 0.05). The pH value of the LP and LB treatments was significantly lower than that of the control after 60 days of ensiling (4.42 and 4.56 vs. 4.71, *p <* 0.05). The LP treatment promoted lactic acid accumulation and LAB number compared with the control (66.59% vs. 54.12% and 8.71 log_10_ CFU/g vs. 8.52 log_10_ CFU/g, respectively, *p <* 0.05), and the LB and LPLB treatments inhibited the growth of yeast and mold after 14 days of fermentation. After 5 days of aerobic exposure, both the LB and LPLB treatments increased the aerobic stability time and acetic acid content (from 29 to 75 h and 16.14%–48.72%, respectively, *p <* 0.05), inhibited the growth of yeast and mold, and did not detect butyric acid. Additionally, the bacteria community of each treatment was dominated by *Aerococcus* on day 3 of ensilage (accounting for 54.36%–69.31%), while the inoculated treatments reduced the abundance of *Aerococcus* on day 60 (from 59.73% to 85.16%, *p <* 0.05), and *Lactobacillus* became the dominant genus (accounting for 54.57%–70.89%). Inoculation of *L. plantarum* effectively maintained the acidic environment at the end of the fermentation system by maintaining the abundance of *Lactobacillus*, maximizing the preservation of dry matter and protein, and reducing protein corruption. Inoculation of *L. brevis* alone or in combination with *L. plantarum* significantly inhibited the growth of mold and improved the aerobic stability of paper mulberry silage.

## Introduction

Paper mulberry (*Broussonetia papyrifera*) is a perennial deciduous tree widely distributed in different areas, such as China, Thailand, and the United States (Saito et al., 2009). In China, it is mainly distributed in the Yellow River, Yangtze River, and Pearl River basins. Paper mulberry has strong adaptability and drought and barrenness resistance. Its leaves are rich in crude protein (CP), crude fat, phenolics, flavonoids, terpenes, and glycosides, and less in fiber (Han et al., 2016). Moreover, it also has the characteristics of cutting resistance and fast growth (Si et al., 2018). Previous studies have shown that adding paper mulberry to the diets of dairy cows, beef cattle, finishing pigs, and goats could save feeding costs; improve livestock production performance, such as meat and milk quality; and enhance the immunity of livestock (Si et al., 2018; Hao et al., 2020; Hua et al., 2020; Tao et al., 2020). However, the characteristics of paper mulberry are similar to those of alfalfa, with high CP content and buffering capacity, which makes it difficult to ensilage directly (Dong et al., 2020). The inoculation of exogenous lactic acid bacteria (LAB) is the main approach used to reduce nutrient loss in silage (Oliveria et al., 2017; Zhang et al., 2018). The application of LAB inoculates prevented spoilage and maximized the preservation of nutrients during ensiling through the regulation of the microbial community of the silage by increasing the number of LAB and improving the types of LAB, thereby ensuring the dominance of LAB during fermentation (Keller et al., 2013; Kharazian et al., 2017). Studies have shown that inoculation of LAB could minimize the loss of nutrients in the leaves of paper mulberry silage (Si et al., 2018; He et al., 2021). However, compared with the exogenous LAB inoculates, the naturally attached LAB strains may have certain advantages in colonization, survival, and growth during ensiling (Ni et al., 2015; Zhao et al., 2020).

The quality of silage fermentation is closely related to microbial succession during the ensiling process. Meanwhile, the microbial community plays an important role in the fermentation process and is likely to be impacted by multiple factors, such as ensiling conditions, inoculants, and epiphytic microorganisms of the fresh forage (Tohno et al., 2012a). However, there is little information available in the epiphytic LAB in paper mulberry. In our previous study, we determined that *Lactiplantibacillus plantarum* and *Lactiplantibacillus brevis* were the excellent LAB in paper mulberry silage (Zhang et al., 2022). Because of the different forms of glucose fermentation, *L. plantarum* and *L. Brevis* are homofermentative and heterofermentative LAB, respectively. The homofermentative LAB produce lactic acid (LA) mainly through glycolysis. The use of this type of inoculant can ensure rapid fermentation during the early stages of ensiling and minimize the loss of nutrients and dry matter (DM; Weinberg and Muck, 2010). The heterofermentative LAB mainly produces LA and acetic acid (AA) through the phosphoketolase pathway. Notably, the capacity of the heterofermentative LAB to produce acid was lower than that of the homofermentative LAB, and the latter also produce CO_2_ during the fermentation process, which increases the loss of nutrients. However, the heterofermentative LAB could produce AA that inhibits mold growth, thereby reducing secondary fermentation and improving the aerobic stability of silage (Filya, 2003; Reich and Kung, 2010). The effect of the dominant LAB (*L. plantarum* and *L. Brevis*) attached to paper mulberry as an inoculant on silage characteristics is not clear.

The present study aims to investigate the effects of *L. plantarum* and *L. brevis* on the fermentation, aerobic stability and bacterial communities composition of paper mulberry silage under laboratory conditions. We hypothesized that it would be feasible to improve fermentation quality and aerobic stability by inoculated with *L. plantarum* and *L. brevis*, and inhibit the growth of undesirable microbial.

## Materials and methods

### Materials and silage preparation

The two LAB strains used in this study were isolated from naturally fermented paper mulberry. The sequences of the strains are deposited in the GenBank database with the following accession numbers: *L. plantarum* MZ008357 and *L. brevis* MZ008360. Previous research has shown that the two LAB strains could grow at different conditions (4–45°C, pH of 3–9, and 35 and 60 g/L of NaCl at 37°C). The theoretical pH values of *L. plantarum* and *L. brevis* were 4.01 and 4.55, respectively, and the OD_600_ values were 1.97 and 1.70, respectively, at 24 h in a liquid medium (Zhang et al., 2022).

The paper mulberry was harvested at 1.5 m plant height in September 2021 from the forages planting demonstration base, Shihezi, Xinjiang Province, China (a temperate desert arid climate, 44.87°N, 85.83°E, and at an altitude of 335 m above sea level). These raw materials were chopped into an approximate length of 1–2 cm with a manual forage chopper and immediately taken to the laboratory. The following treatments were applied before ensilage: (1) control (addition of sterilized water 10 ml/kg); (2) inoculation with *L. plantarum* at 5 × 10^6^ colony forming units (CFU/g) of wet silage (LP treatment); (3) inoculation with *L. brevis* at 5 × 10^6^ CFU/g of wet silage (LB treatment); and (4) inoculation with the *L. plantarum* and *L. brevis* mixed at a ratio of 1:1 at 5 × 10^6^ CFU/g of wet silage (LPLB treatment). Each LAB strain was enriched with the de Man Rogosa Sharpe liquid medium. After the number of LAB was determined by counting the plate, the bacteria were evenly sprayed on the per bag of silage surface and completely mixed for storage. A total of 100 vacuum-sealed polyethylene plastic bags (dimensions 35 cm × 40 cm) were prepared with silage, five bags per treatment (approximately 1 kg), and ensiled at ambient temperature (24°C–40°C). Each treatment was randomly opened in three bags during the experimental period (3, 7, 14, 30, and 60 days), and samples were taken per bag for measuring the indicators.

### Chemical components, fermentation characteristics, and microbiological analyses

The chemical components, fermentation characteristics, and microbiological analyses were performed in triplicate. The DM content of the fresh material and silage was determined by oven drying for 48 h at 60°C (AOAC, 1990), pulverized to pass through a 1-mm screen using a plant crusher (DFY-1000D, Linda Instrument Co., Ltd., Zhejiang, China). The neutral detergent fiber (NDF) and acid detergent fiber (ADF) were measured using the Van Soest method (Soest, 1963). The CP content was determined using the Kjeldahl method (AOAC, 1990). Fresh silage sample of 20 g mixed with 180 ml distilled water and stored at 4°C for 24 h, then filtered through four layers of gauze. The pH value was measured using a pH meter (PHS-3C, Instrument and Electrical Science Instrument Co., Ltd., Shanghai, China). The ammoniacal nitrogen (NH_3_-N) was analyzed using the phenol-hypochlorite procedure described by Weatherburn (1967). The water soluble carbohydrates (WSCs) were determined using the anthrone colorimetry (Murphy, 1958). The concentration of organic acids were measured using high-performance liquid chromatography (Agilent Technologies, Inc., Waldbronn, Germany) as described by Yang et al. (2020), using a C18 column (150 mm × 4.6 mm) with an oven temperature of 50°C and flow rate of 0.6 ml/min.

Take 10 g silage samples from each bags blended with 90 ml sterilized saline water (8.5 g/L NaCl) and serially diluted. The number of LAB, yeasts, molds, and aerobic bacteria (AB) was enumerated on de Man Rogosa Sharpe agar, Malt Extract Agar for yeast, Salt Czapek Dox Agar and Plate Count Agar, respectively (Land Bridge Technology Co., Ltd., Beijing, China).

### Aerobic stability analysis

After 60 days of ensiling, the silages were subjected to a 5-day aerobic stability test at 24.0°C ± 1.0°C described by Tabacco et al. (2009). In this test, the pH, content of organic acid, number of LAB, yeasts, molds, and AB serve as spoilage indicators. Aerobic stability time was defined as the time until the internal temperature of the silage exceeded the environmental temperature by 2°C (Schmidt and Kung, 2010) as determined by a multipoint real-time temperature recorder (model I500-E3TW, Yuhuan Zhituo Instrument Technology Co. Ltd., China).

### Bacterial community analyses

Total DNAs were extracted using a bacterial DNA Kit D3350-02 (Omega Biotek, Norcross, GA, United States) according to the manufacturer’s protocols. Primers targeting the V3-V4 regions of 16S rDNA 338F: ACTCCTACGGGAGGCAGCAG; 806R: GGACTACHVGGGTWTCTAAT (Li et al., 2017). The amplicons were extracted, purified and analyzed following the method describe by Amato et al. (2013). The quality-filter, cluster, and analysis for 16S rRNA sequencing data were performed as described by Yu et al. (2016). The sequence data reported in this study have been deposited in the NCBI database (Accession No. PRJNA845912).

### Statistical analyses

The effects of ensiling time and LAB on the fermentation products of paper mulberry silage were statistically analyzed by IBM SPSS 20.0 (IBM, Chicago, IL, United States). The results were evaluated using two-way analysis of variance (ANOVA), with Duncan’s multiple range test. A *p*-value of <0.05 was considered significantly different. The bacterial relative abundance and its correlation with several different fermentation parameters were analyzed on the free online Majorbio Cloud Platform.^1^

## Results

### Characteristics of paper mulberry before ensiling

The paper mulberry had a DM of 361.1 g/kg fresh matter (FW), CP of 20.84% DM, NDF of 43.80% DM, ADF of 20.15% DM, WSC of 10.54% DM, LAB of 7.18 log_10_ CFU/g FM, yeast of 6.54 log_10_ CFU/g FM, mold of 5.30 log_10_ CFU/g FM, and AB of 7.16 log_10_ CFU/g FM.

### Chemical components, fermentation characteristics, and microbial population in paper mulberry silage

The dynamics of chemical components of paper mulberry silage inoculated with *L. plantarum* and/or *L. brevis* are shown in Table 1. The DM content of LB treatment was significantly lower than that of control and LP treatments on day 3 of ensiling (*p* < 0.05) but significantly higher than that of LP treatment on day 30 of ensiling (*p* < 0.05). After the 60-day of ensiling, the DM content in LP treatment was significantly higher than in the control (*p* < 0.05). The content of CP in control was significantly lower than that in LP treatment on day 14 of ensiling (*p* < 0.05), and the LB treatment showed significantly higher CP content than that of the other treatments after the 60 days of ensilage (*p* < 0.05). In comparison to the control silage, the significantly lower NDF content were found in the inoculated treatments on day 3 and 60 (*p* < 0.05), and the LP treatment showed significantly lower ADF content than control after 3 days of ensilage (*p* < 0.05).

**Table 1:** Effects of *Lactiplantibacillus plantarum* and *Lactiplantibacillus brevis* on chemical compositions of paper mulberry silage.

Days	Treatment^1^	DM	CP	NDF	ADF
3	Control	36.77^Aa^	19.86^Aa^	43.49^Aa^	21.51^Aa^
	LP	37.13^Aa^	19.28^ABa^	38.93^Ab^	18.41^Bb^
	LB	35.77^Ab^	19.72^Aa^	39.26^Ab^	20.44^Aab^
	LPLB	36.66^Aab^	19.37^Aa^	39.40^Ab^	19.56^Aab^
	SEM	0.138	0.134	0.611	0.324
	*P-*value	0.044	0.398	0.088	0.049
14	Control	35.04^Ba^	18.60^ABb^	40.34^Aa^	21.47^Aa^
	LP	35.46^Ca^	19.48^Aa^	40.70^Aa^	20.82^Aa^
	LB	35.65^Aa^	19.14^ABab^	38.56^Aa^	22.52^Aa^
	LPLB	35.73^Aa^	19.16^Aab^	36.06^Aa^	21.57^Aa^
	SEM	0.113	0.111	0.151	0.502
	*P-*value	0.224	0.016	0.696	0.695
30	Control	36.41^Aab^	17.5^Ba^	39.27^Aa^	20.08^Aa^
	LP	36.85^ABa^	18.56^Ba^	39.13^Aa^	19.01^Ba^
	LB	35.46^Ab^	18.27^ABa^	38.34^Aa^	20.41^Aa^
	LPLB	36.42^Aab^	18.78^Aa^	37.52^Aa^	21.98^Aa^
	SEM	0.127	0.386	0.793	0.455
	*P-*value	0.014	0.67	0.853	0.22
60	Control	35.01^Bb^	17.88^Bb^	42.78^Aa^	20.16^Aa^
	LP	36.10^BCa^	18.52^Ba^	38.91^Ab^	21.04^Aa^
	LB	35.35^Aab^	17.22^Bb^	38.46^Ab^	20.68^Aa^
	LPLB	35.81^Aab^	17.50^Bb^	39.64^Ab^	19.33^Aa^
	SEM	0.139	0.102	0.319	0.872
	*P-*value	0.024	0.009	0.005	0.907
*P-*value	Day	**	*	NS	NS
	Treatment	NS	NS	**	NS
	D × T	**	NS	NS	NS

Means of three observations. Different lowercase letters indicate significant differences (*P* < 0.05) between different treatments at the same time. Different capital letters indicate significant differences (*p* < 0.05) between the different times of the same treatment. DM, dry matter; CP, crude protein; NDF, neutral detergent fiber; ADF, acid detergent fiber. Percentage of fresh weight for DM. Percentage of DM for CP, NDF, and ADF.

1 Control, ensiling with no inoculant applied; LP, inoculated with *L. plantarum* MZ008357; LB, inoculated with *L. brevis* MZ008360; LPLB, inoculated with *L. plantarum* and *L. brevis*; SEM, standard error of means.

* *p* < 0.05;

** *p* < 0.01.

The dynamics of fermentation characteristics of paper mulberry silage are presented in Table 2. The ensiling days, inoculants and their interaction significantly affected the pH, WSCs and concentration of organic acid (*p* < 0.01). The pH decreased slowly in each treatment within 60 days of ensilage. The silage in the control had a higher pH than that of the inoculated treatments on day 14 of ensilage (*p* < 0.05). The LP treatment showed significantly lower pH than that of the LB treatment on day 3 and 60 of ensiling (*p* < 0.05), both LP and LB treatments significantly decreased the pH after 60 days of ensiling (*p* < 0.05). In comparison to the control silage, the significantly higher NH_3_-N content were found in the inoculated treatments on day 3 of ensilage (*p* < 0.05), however, the significantly lower NH_3_-N content were found in the inoculated treatments after 60 days (*p* < 0.05). Of these, the LP treatment significantly decreased the NH_3_-N content compared to the LB and LP treatments (*p* < 0.05). The WSCs content of the control silage was always at a high level within 60 days of fermentation, and significantly higher than the inoculated silages on day 14 and 60 of ensilage (*p* < 0.05). The LA concentration gradually increased during silage fermentation, and the LP treatment was significantly increased the LA concentration compared with the other treatments on day 14 and 60 of ensiling (*p* < 0.05). The LB treatment showed significantly higher AA concentration than that of the control and LP treatment on day 3 and 30 of ensiling (*p* < 0.05), and the LPLB treatment was always showed significantly higher AA concentration than that of the LP treatment during 14–30 days of ensilage (*p* < 0.05). The significantly higher PA concentration were found in the LB treatment compared to the control and LP treatment on day 3 of ensilage (*p* < 0.05), however, the significantly lower PA concentration were found in the inoculated silages compared with the control after 60 days of ensilage (*p* < 0.05). BA was not detected throughout the fermentation.

**Table 2:** Effects of *Lactiplantibacillus plantarum* and *Lactiplantibacillus brevis* on fermentation characteristics of paper mulberry silage.

Days	Treatment^1^	pH	NH_3_-N	WSCs	LA	AA	PA
3	Control	5.70^Aa^	0.07^Db^	8.95^Aa^	16.34^Dd^	6.33^Cc^	0.73^Dc^
	LP	5.27^Ac^	0.10^Da^	9.05^Aa^	26.21^Db^	14.61^Bb^	3.24^Bb^
	LB	5.55^Ab^	0.11^Da^	8.94^Aa^	23.50^Cc^	17.50^Ba^	4.07^Aa^
	LPLB	5.27^Ac^	0.11^Da^	9.21^Aa^	29.12^Ca^	15.07^Cb^	3.73^Aab^
	SEM	0.021	0.003	0.071	0.164	0.333	0.103
	*P-*value	<0.001	<0.001	0.508	<0.001	<0.001	<0.001
14	Control	5.19^Ba^	0.32^Ca^	8.63^ABa^	32.69^Cbc^	15.24^Bab^	3.96^Aa^
	LP	4.84^Bb^	0.21^Cb^	7.60^Bb^	38.79^Ca^	14.87^Bb^	4.31^Aa^
	LB	4.86^Bb^	0.23^Cb^	7.73^Bb^	27.74^Cc^	16.45^Bab^	3.95^Aa^
	LPLB	4.88^Bb^	0.35^Ca^	7.22^Bb^	33.45^Cb^	20.24^Ba^	4.50^Aa^
	SEM	0.012	0.005	0.089	0.76	0.799	0.172
	*P-*value	<0.001	<0.001	0.001	0.006	0.141	0.629
30	Control	5.15^Ba^	0.42^Ba^	8.33^Ba^	46.84^Ba^	19.06^Ab^	3.08^Ba^
	LP	4.87^Ba^	0.36^Ba^	7.45^Bb^	47.97^Ba^	19.86^Ab^	2.74^Ba^
	LB	4.82^Ba^	0.42^Ba^	7.86^Bab^	42.04^Ba^	27.36^Aa^	2.28^Ba^
	LPLB	4.85^Ba^	0.37^Ba^	6.86^Bc^	47.14^Ba^	26.47^Aa^	2.48^Ba^
	SEM	0.01	0.01	0.087	0.908	0.805	0.122
	*P-*value	<0.001	0.116	<0.001	0.168	0.011	0.189
60	Control	4.71^Ca^	0.65^Aa^	7.42^Ca^	54.12^Ab^	17.26^ABa^	2.34^Ca^
	LP	4.42^Cc^	0.46^Ac^	6.11^Cb^	66.59^Aa^	18.87^Aa^	1.44^Cc^
	LB	4.56^Cb^	0.51^Ab^	6.30^Bb^	62.69^Aab^	21.27^Ba^	1.22^Cc^
	LPLB	4.63^Cab^	0.51^Ab^	6.77^Bb^	55.17^Aab^	20.71^Ba^	1.82^Bb^
	SEM	0.013	0.006	0.104	1.794	0.651	0.054
	*P-*value	<0.001	<0.001	0.004	0.109	0.165	<0.001
*p-*value	Day	**	**	**	**	**	**
	Treatment	**	**	**	**	**	*
	D × T	**	**	**	**	*	**

Means of three observations. Different lowercase letters indicate significant differences (*P* < 0.05) between different treatments at the same time. Different capital letters indicate significant differences (*P* < 0.05) between the different times of the same treatment. WSCs, water-soluble carbohydrates; NH_3_-N, ammoniacal nitrogen; LA, lactic acid; AA, acetic acid; PA, propionic acid. Percentage of DM WSCs. Percentage of total nitrogen for NH_3_-N; g/kg of DM for LA, AA, and PA.

1 Control, ensiling with no inoculant applied; LP, inoculated with *L. plantarum* MZ008357; LB, inoculated with *L. brevis* MZ008360; LPLB, inoculated with *L. plantarum* and *L. brevis*; SEM, standard error of means.

* *p* < 0.05;

** *P* < 0.01.

The dynamics of the numbers of the main microorganisms of paper mulberry silage are presented in Table 3. The effects of ensiling days, inoculants and their interaction on the silage main microorganisms were significant (*p* < 0.01). The number of LAB, yeast, and AB decreased during silage fermentation. In comparison to the LB treatment, the significantly higher LAB count were found in the LP treatment after ensilage for 3 days (*p* < 0.05), and the significantly lower LAB count were found in the control and LPLB treatment on day 14 of ensilage (*p* < 0.05). In addition, the LPLB treatment showed significantly higher LAB counts than that other treatments during 30–60 days of ensiling (*p* < 0.05). The significantly lower yeast count were found in the LPLB treatment compared with the control during ensilage (*p* < 0.05), and all the inoculated treatments also showed significantly lower yeast count than that of the control after 60 days of ensilage (*p* < 0.05). On day 14 of ensilage, the significantly higher mold number were found in the control than that in the inoculated silage (*p* < 0.05). However, the mold was only present in the control and LP treatment during the 30–60 days of ensiling. The LPLB treatment showed significantly higher AB count than that of the other treatments on day 3 of ensiling (*p* < 0.05), and the silage in the control and LPLB treatment had a higher AB count than that of the LP and LB treatment during the 30–60 days of ensilage (*p* < 0.05).

**Table 3:** Effects of *Lactiplantibacillus plantarum* and *Lactiplantibacillus brevis* on microbial population of paper mulberry silage.

Days	Treatment^1^	LAB	Yeast	Mold	AB
3	Control	9.46^Ac^	5.03^Aa^	3.80^Ba^	9.09^Ac^
	LP	9.73^Aa^	4.89^Aab^	3.92^Aa^	9.21^Ab^
	LB	9.57^Abc^	4.78^Ab^	3.91^Aa^	9.19^Ab^
	LPLB	9.65^Aab^	4.55^Bc^	3.86^Aa^	9.37^Aa^
	SEM	0.018	0.027	0.021	0.006
	*P-*value	0.004	0.001	0.218	<0.001
14	Control	8.45^Bc^	4.84^ABa^	4.14^Aa^	8.38^Bb^
	LP	9.57^Aa^	4.80^Aab^	3.87^Ab^	8.45^Bb^
	LB	9.56^Aa^	4.58^Bc^	3.70^Ac^	8.66^Ba^
	LPLB	9.34^Bb^	4.71^Abc^	3.53^Bd^	8.35^Bb^
	SEM	0.024	0.023	0.01	0.022
	*P-*value	<0.001	0.009	<0.001	0.004
30	Control	8.50^Bc^	4.54^Ba^	3.77^Ba^	8.36^Ba^
	LP	8.97^Bb^	4.41^Bb^	3.62^Bab^	8.11^Cb^
	LB	9.03^Bab^	4.27^Cc^	<2.00	8.13^Cb^
	LPLB	9.23^Ca^	4.33^Cbc^	<2.00	8.33^Ba^
	SEM	0.036	0.015	0.025	0.021
	*P-*value	0.001	0.002	0.076	0.005
60	Control	8.52^Bc^	3.80^Ca^	4.15^Aa^	7.86^Ca^
	LP	8.71^Cb^	3.72^Cab^	3.71^Cb^	7.53^Db^
	LB	8.60^Cc^	3.54^Dc^	<2.00	7.40^Db^
	LPLB	8.88^Da^	3.62^Dbc^	<2.00	7.71^Ca^
	SEM	0.016	0.025	0.053	0.023
	*P-*value	<0.001	0.029	0.022	0.001
*P-*value	Day	**	**	**	**
	Treatment	**	**	**	**
	D × T	**	**	**	**

Means of three observations. Different lowercase letters indicate significant differences (*P* < 0.05) between different treatments at the same time. Different capital letters indicate significant differences (*P* < 0.05) between the different times of the same treatment. LAB, lactic acid bacteria; AB, aerobic bacteria. Percentage of Log_10_ CFU/g of fresh matter for LAB, yeast, mold, and AB.

1 Control, ensiling with no inoculant applied; LP, inoculated with *L. plantarum* MZ008357; LB, inoculated with *L. brevis* MZ008360; LPLB, inoculated with *L. plantarum* and *L. brevis*; SEM, standard error of means.

* *p* < 0.05;

** *P* < 0.01.

### Aerobic stability of paper mulberry silage

The results of the aerobic test (5 days) are shown in Table 4. After the paper mulberry silage was unpacked for 5 days, except for LP treatment, other inoculated treatments significantly improved the aerobic stability time compared with the control (*p <* 0.05). The LP and LB treatments showed significantly higher pH than that of the control (*p <* 0.05). The LA concentrations was in the order of LP > LPLB > control > LB (*p <* 0.05), and the AA was ranked as LB > LPLB > LP > control (*p <* 0.05). The significantly lower PA concentration were found in the inoculated silages compared with the control (*p <* 0.05), and the LP and LPLB treatments showed significantly higher PA concentration than that in LB treatment (*p <* 0.05). The BA concentration of the control was significantly higher than that of the LP treatment (*p <* 0.05), and the other treatments were not detected.

**Table 4:** Effect of inoculation of *Lactiplantibacillus plantarum* and *Lactiplantibacillus brevis* on the aerobic stability of paper mulberry silage.

Items	Treatment^1^				SEM	*P*-value
	Control	LP	LB	LPLB		
Aerobic stability time	63^c^	59^c^	134^a^	92^b^	13.5	<0.001
pH	5.12^c^	5.24^a^	5.20^ab^	5.15^bc^	0.007	0.002
LA	34.2^c^	48.2^a^	30.4^d^	38.4^b^	0.579	<0.001
AA	10.55^c^	12.02^c^	15.69^a^	13.96^b^	0.233	<0.001
PA	5.78^a^	5.05^b^	3.75^c^	4.86^b^	0.07	<0.001
BA	3.3^a^	0.8^b^	ND	ND	0.011	<0.001
LAB	8.13^c^	8.64^a^	8.56^b^	8.62^a^	0.009	<0.001
Yeast	4.52^a^	4.48^a^	4.28^b^	4.51^a^	0.012	<0.001
Mold	4.26^a^	4.16^b^	3.93^c^	3.84^d^	0.006	<0.001
AB	9.05^a^	8.38^c^	8.40^c^	8.64^b^	0.01	<0.001

Means of three observations. Different lowercase letters indicate significant differences (*P* < 0.05) between different treatments at the same time. LA, lactic acid; AA, acetic acid; LA/AA, lactic acid/acetic acid; PA, propionic acid; LAB, lactic acid bacteria; AB, aerobic bacteria. h for aerobic stability time; g/kg of DM for LA, AA, and PA. Log_10_ CFU/g of fresh matter for LAB, yeast, mold, and AB.

1 Control, ensiling with no inoculant applied; LP, inoculated with *L. plantarum* MZ008357; LB, inoculated with *L. brevis* MZ008360; LPLB, inoculated with *L. plantarum* and *L. brevis*; SEM, standard error of means.

The inoculated treatments showed significantly higher LAB number than that of the control (*p <* 0.05), and the significantly lower LAB number were found in LB treatment compared to the LP and LPLB treatments (*p <* 0.05). The LB treatment significantly decreased the yeast number compared to the other treatments (*p <* 0.05). and the inoculated treatments were significantly decreased the mold and AB counts (*p <* 0.05), among which the mold count in the LPLB treatment were the lowest (*p <* 0.05), and the AB count in the LP treatment was the lowest (*p <* 0.05).

### Bacterial community composition in paper mulberry silage

**Figure 1:**
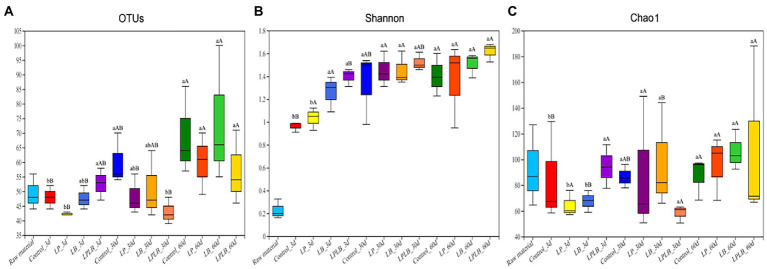
Box-plots of observed bacterial species **(A)**, Shannon **(B),** and Chao1 indices **(C)** of bacterial communities in paper mulberry silage. Control, ensiling with no inoculant applied; LP, inoculated with *Lactiplantibacillus plantarum*; LB, inoculated with *Lactiplantibacillus brevis*; LPLB, inoculated with *L. plantarum* and *L. brevis*. OTUs, observed bacterial species. Different lowercase letters indicate significant differences (*p <* 0.05) between different treatments at the same time. Different capital letters indicate significant differences (*p <* 0.05) between the different time at the same treatment.

Bacterial diversity of paper mulberry silage was determined by high-throughput analysis. A total of 1,746,473 optimized sequences were obtained, and the valid sequences were clustered into 266 OTUs based on 97% sequence identity. Figures 1A–C showed the dynamics of Shannon and Chao1 indices. The microbial diversity increased in the control and LPLB treatment after ensiling compared with that in the 3 and 30 day of ensilage (*p <* 0.05); however, it did not change markedly in the silage of the LP and LB treatments after 60-day ensilage (*p* > 0.05). The significantly lower microbial richness were found in the LP and LB treatments compared to the control on day 3 of ensiling (*p <* 0.05), and the microbial richness of LB treatment increased after the 60-day ensilage compared to the 3 days (*p <* 0.05).

**Figure 2:**
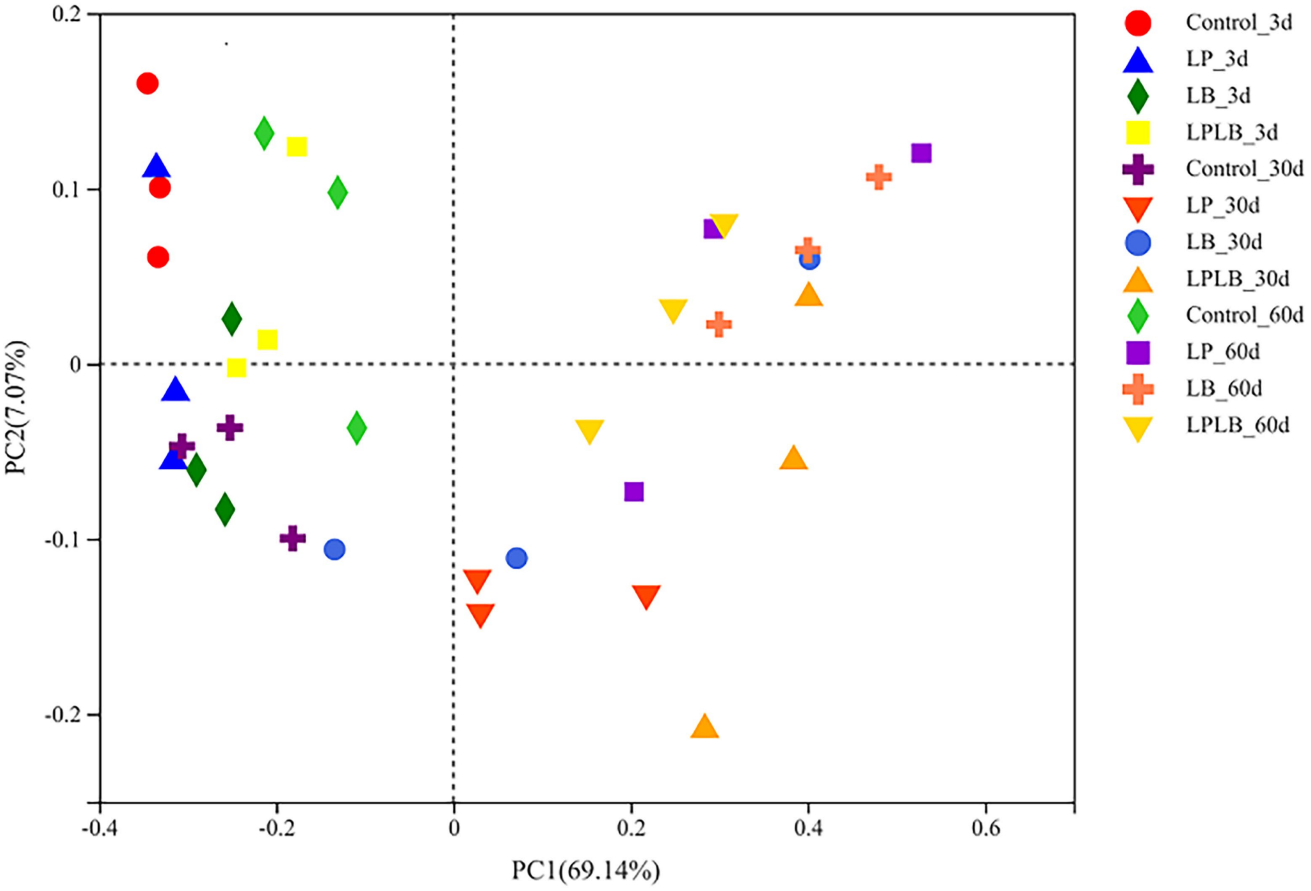
Principal-coordinate analysis (PCoA) plots based on weighted UniFrac distance for the bacterial community. Control, ensiling with no inoculant applied; LP, inoculated with *Lactiplantibacillus plantarum*; LB, inoculated with *Lactiplantibacillus brevis*; LPLB, inoculated with *L. plantarum* and *L. brevis*.

In addition, using the principal coordinate analysis (PCoA) to assess the bacterial communities (Figure 2), the results indicate that the inoculation of *L. plantarum* and *L. brevis* shifted the distribution of the bacterial community on day 30 and 60 of ensilage (*R* = 0.6672, *p <* 0.01).

**Figure 3:**
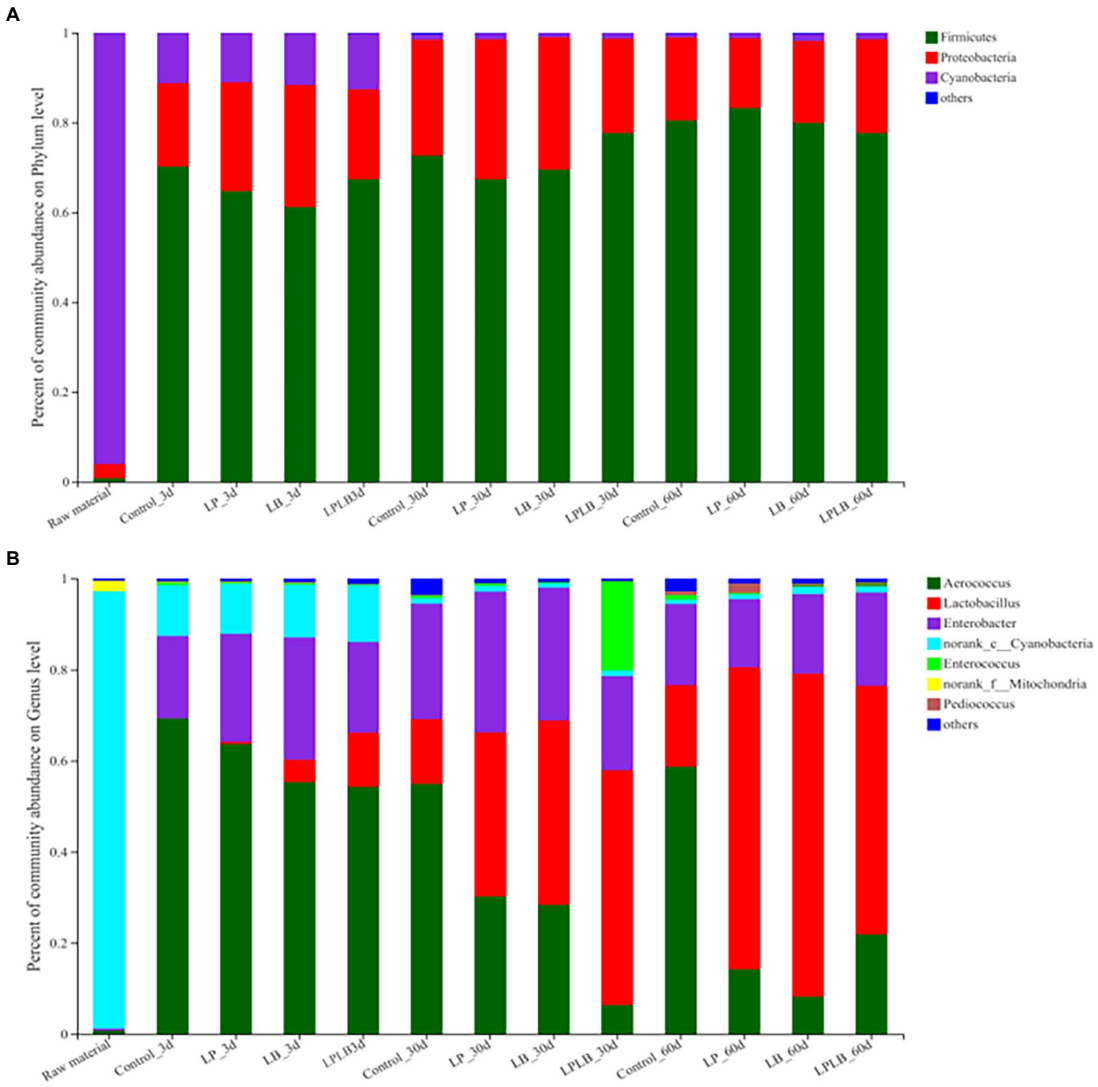
Bacterial community and relative abundances by phylum **(A)** and genus **(B)** for paper mulberry silage. Control, ensiling with no inoculant applied; LP, inoculated with *Lactiplantibacillus plantarum*; LB, inoculated with *Lactiplantibacillus brevis*; LPLB, inoculated with *L. plantarum* and *L. brevis*.

Cyanobacteria (95.93%) was the predominant phylum in the raw material (Figure 3A). Firmicutes became the predominant phylum after ensiling, followed by Proteobacteria. At the genus level, norank_c_*Cyanobacteria* (95.93%) dominated the epiphytic bacterial community of paper mulberry, followed by norank_f_*Mitochondria*. After 60 days of ensiling, four genera, namely *Aerococcus*, *Lactobacillus*, *Enterobacter*, and *Enterococcus*, had an average relative abundance of more than 1%, and their relative abundances were 30.77%, 38.80%, 20.10%, and 2.06%, respectively. These four genera account for 91.73% relative abundance of the bacterial community (Figure 3B). On day 3 of ensiling, the dominant genus was *Aerococcus*, followed by *Enterobacter*; the relative abundances of *Aerococcus* in the control (69.31%) and LP treatment (63.8%) were the highest (*p* < 0.05). The relative abundance of *Lactobacillus* in the LPLB treatment was the highest with 11.91%. The *Aerococcus* (54.94%) was the dominant genus of the control silage in contrast to the *Lactobacillus* for the inoculated silage after 30 days of ensilage, and highest abundance of *Lactobacillus* (51.55%) present in the LPLB treatment. On day 60 of ensilage, the dominant genus of the control was *Aerococcus* (58.71%), followed by *Lactobacillus* (17.88%). *Lactobacillus* was predominant in the inoculated treatments after 60 days of ensilage (54.57–70.89%), and the relative abundance of *Lactobacillus* in the LB treatment was the highest (70.89%). The relative abundance of *Enterobacter* during the entire ensiling process was not significantly different between the control and the inoculated treatments (14.94%–30.89%).

**Figure 4:**
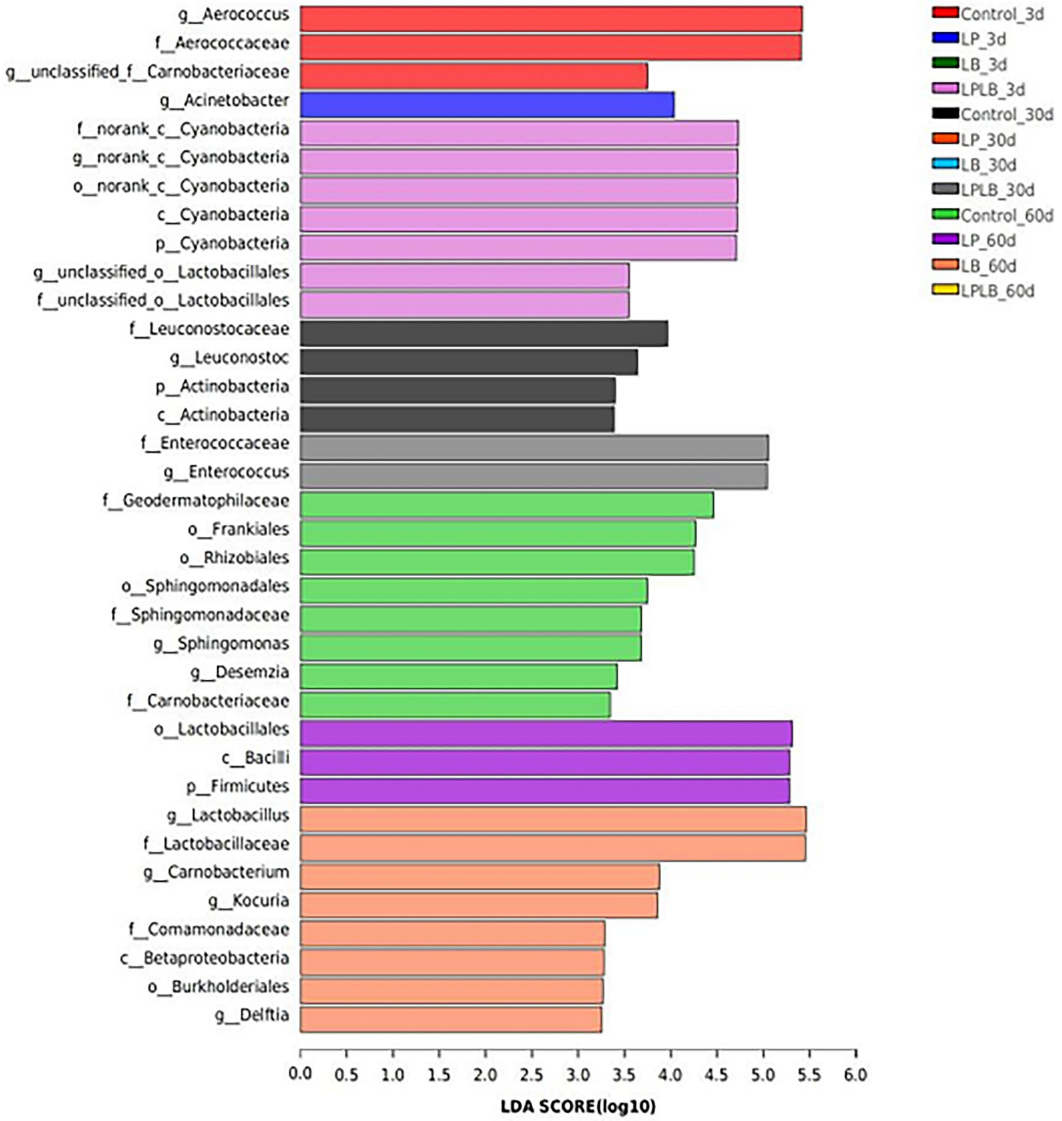
Comparison of microbial variations using LefSe analysis of paper mulberry silage. Control, ensiling with no inoculant applied; LP, inoculated with *Lactiplantibacillus plantarum*; LB, inoculated with *Lactiplantibacillus brevis*; LPLB, inoculated with *L. plantarum* and *L. brevis*.

LefSe was performed to further explore the variations in the bacterial communities among the treatments (Figure 4). A significantly higher relative abundance of *Aerococcus* was observed in the control on day 3 of ensiling (*p* < 0.05). The LPLB treatment showed significantly higher abundance of *Enterococcus* on day 30 of ensiling (*p* < 0.05). *Lactobacillus* grew well in the LB treatment on day 60 of ensiling.

**Figure 5:**
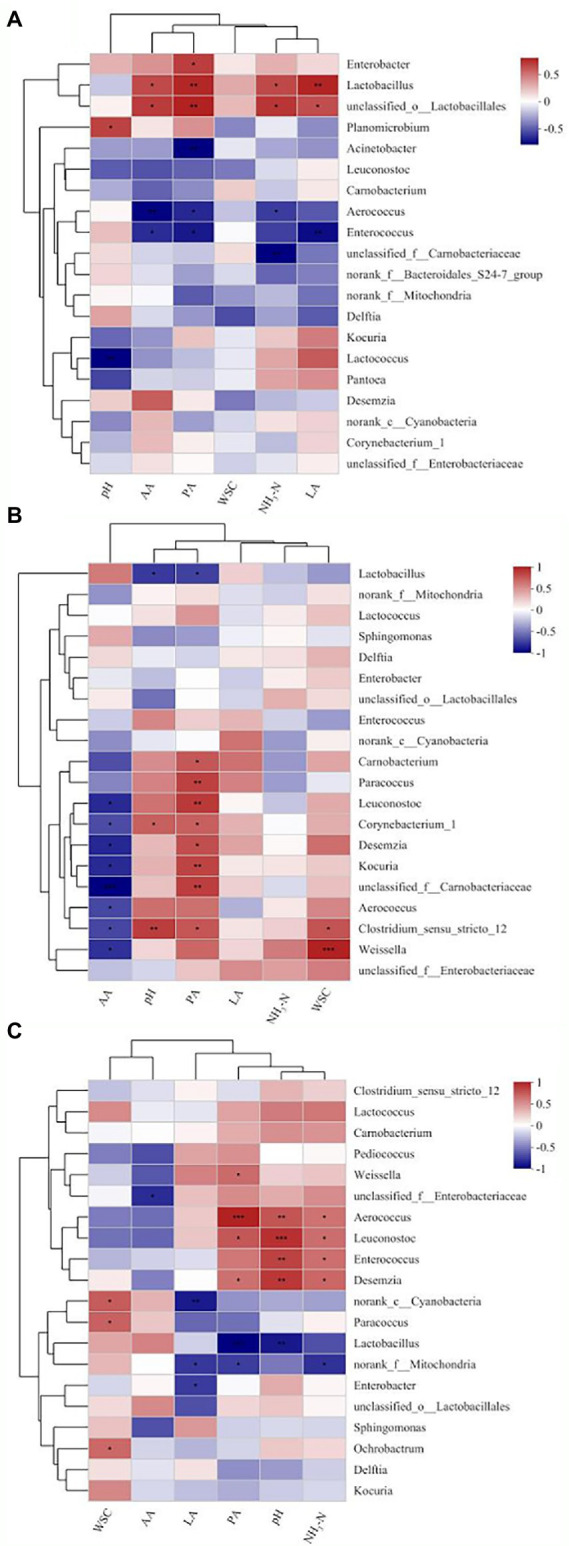
Spearman’s correlation heatmap of the abundance of bacterial genera and fermentation properties in paper mulberry ensiled for 3 days **(A)**, 30 days **(B)**, and 60 days **(C)**. ^*^p < 0.05, ^**^p < 0.01 and ^***^p < 0.001.

Spearman’s correlation heatmap further illustrated the correlations between contents of metabolites and microbial kinetics for different periods (Figures 5A–C). *Lactobacillus* was significantly positively correlated (*p <* 0.01) with the concentration of LA (*R* = 0.73) and PA (*R* = 0.72), and negatively correlated (*p <* 0.05) with the concentration of AA (*R* = −0.60) and NH_3_-N (*R* = −0.61) on day 3 of ensilage. *Lactobacillus* was negatively correlated (*p <* 0.05) with pH (*R* = −0.64) and the concentration of PA (*R* = −0.62) from 30–60 days of ensilage. Negative correlations (*p <* 0.05) were found between *Aerococcus* and the concentration of AA (*R* = −0.74 on day 3 and − 0.59 on day 30). *Aerococcus* was negatively correlated (*p <* 0.05) with the concentration of PA (*R* = −0.64) and NH_3_-N (*R* = −0.59) on day 3, significantly positively correlated (*p <* 0.01) with the concentration of PA (*R* = 0.91) and pH (*R* = 0.71), and positively correlated (*p <* 0.05) with NH_3_-N (*R* = 0.59) on day 60 of ensilage. *Enterobacter* was positively correlated (*p <* 0.05) with the concentration of PA (*R* = 0.64) on day 3 and negatively correlated (*p <* 0.05) with the concentration of LA (*R* = −0.64) on day 60. The relative abundances of the other genera were low (<1%), hence not analyzed.

## Discussion

### Fermentation properties in paper mulberry silage

A series of biochemical changes occur in silage due to the action of a variety of microorganisms and the enzyme system of plants during the silage process, leading to the loss of nutrients. In the present study, inoculation of *L. brevis* only reduced the DM content at the early stage (3 days) of paper mulberry silage fermentation, and the consumption of DM reduced gradually with the prolongation of fermentation time. It was similar to the results of the study by Bai et al. (2020). The DM content of the fermentation system inoculated with *L. plantarum* at the end of fermentation (60 days) was significantly higher than that of the control, and similar results were observed by Ren et al. (2020). It has been shown that inoculation with *L. plantarum* effectively promotes homofermentation and inhibits organic degradation due to insufficient LA production (Ren et al., 2020). Research has shown that an adequate carbon source (WSC content of at least 5% DM) is required for silage fermentation (Ni et al., 2017). In the present study, the WSC content of fresh paper mulberry was 10.54%. With the progress of fermentation, the inoculation treatments consumed a large amount of WSC, resulting in its content being significantly reduced to below 7% after fermentation. The consumption of WSC resulting in the number of LAB in each inoculation treatment rapidly became the dominant flora in the early stage of silage fermentation, promoting the formation of an anaerobic and acidic environment until the end of the fermentation quantity tends to be stable, the consumption of WSC is also basically constant. Inoculation of LAB in silage usually consumes more WSC, which is converted to organic acids (such as LA and AA) to maintain the acidic environment of the fermentation system. However, there is currently a controversy over how the inoculation of *L. brevis* affects the pH of the fermentation system (Kung et al., 2003;Xu et al., 2017). In the present study, inoculation with *L. brevis* significantly changed the acidic environment of paper mulberry silage, but it was weaker than inoculation with *L. plantarum*. At the early stage of silage fermentation, inoculation of *L. plantarum* alone or in combination with *L. brevis* could reduce the pH value by increasing the number of LAB and producing a large amount of LA in the fermentation system. At the end of the silage fermentation, inoculation of *L. plantarum* and *L. brevis* significantly reduced the pH of the fermentation system, while the combined inoculation of *L. plantarum* and *L. brevis* maintained the highest number of LAB in the fermentation system but produced inadequate LA to reduce pH. This might be due to the competitive interaction between *L. plantarum* and *L. brevis* for substrates. In addition, inoculation treatments inhibited the growth of mold and AB at the end stage of fermentation. *L. brevis*, whether inoculated alone or in combination with *L. plantarum*, showed the strongest inhibition effect on yeast and mold, further confirming the heterofermentative effect of *L. brevis* (Garde et al., 2002).

The change in the CP content of silage is closely related to the pH value, which affects the activities of protease (carboxypeptidase and acidic protease) in forage (McKersie and Buchanan-Smith, 1982). Protein hydrolysis and decarboxylation of silage are reduced by acidification, which inhibits proteolytic enzyme activity and reduces NH_3_-N content (Wang et al., 2009; Li et al., 2018). In this study, at the end of silage fermentation, each inoculation treatment significantly reduced the NH_3_-N content of the fermentation system, and inoculation of *L. plantarum* could maintain the lowest acidic environment (pH = 4.42) to maximize the preservation of the CP of the silage system, which is in accordance with the results of Ren et al. (2020). Previous research has demonstrated that the main factors affecting protein spoilage in silage are related to the activities of *Enterobacter* and *Clostridium* (McDonald et al., 1991). When the pH value dropped below 4.5, the growth of *Enterobacter* in the silage was significantly inhibited (Muck, 2010). *Clostridium* was not detected in each treatment in this study, so it might be the acidic environment created by the inoculation of *L. plantarum* that exerted strong inhibition on *Enterobacter* and promoted a substantial reduction in protein corruption. Moreover, the acidic environment could also effectively inhibit the growth of a variety of other undesirable microorganisms (e.g., *Enterobacter*, *Bacillus*, and *Acetic acid bacteria*) and the activity of plant protease (Ni et al., 2017). In addition, at the end of fermentation, each treatment could significantly reduce the NDF content of the fermentation system but did not affect the ADF content, which might be due to the effect of hemicellulase. A meta-analysis also showed that inoculation with LAB could significantly reduce the NDF content of silage (Blajman et al., 2020).

### Aerobic stability in paper mulberry silage

The characteristics of silage fermentation are directly related to aerobic deterioration. A previous study on the relationship between the characteristics of silage of 50 species of forage grass and aerobic deterioration showed that LA reduction after aerobic deterioration had a significant negative correlation with pH value and a significant positive correlation with the residual WSCs content (Ohyama et al., 2010). Similar results were obtained in this study. The pH value of each treatment increased to 5.08–5.36, and the concentration of LA and AA significantly decreased (by 38.2%–78.0% and 46.5%–71.7%, respectively) after the aerobic test (5 days). In general, the aerobic microorganisms in silage do not all come from the external environment. The aerobic exposure process creates the appropriate breeding conditions for the latent aerobic microorganisms in the fermentation system (Yu and Sun, 2011), with the decomposition and utilization of LA by assimilating yeast, the pH of the silage increased gradually, and when the pH reached 4.5, the breeding of aerobic bacteria and harmful bacteria intensified (Muck, 2010). Studies have shown that the number of yeasts directly affects the aerobic stability of silage. When the number of yeast exceeds 5 log_10_ CFU/kg FW, the aerobic stability of silage decreases significantly (Wilkinson and Davies, 2013). In the present study, after the aerobic test (5 days), the number of LAB was higher and the numbers of mold and AB were lower in each inoculation treatment compared with control, which was mainly because although the activity of the inoculated LAB was not enough to maintain the aerobic stability for a long time after the silage was opened. It could still inhibit the growth and reproduction of aerobic microorganisms by maintaining the acidity and the production of antibacterial substances in a short time (Wang et al., 2018). Especially, when *L. brevis* was inoculated alone or in combination with *L. plantarum*, a higher AA content could be maintained in an aerobic environment to inhibit the growth of mold and AB and improve the aerobic stability time of paper mulberry silage to the greatest extent.

### Bacterial community in paper mulberry silage

In the present study, *Aerococcus* was the dominant strain for each treatment after 3 days of ensiling, and its relative abundance decreased with prolonged ensiling. *Aerococcus* is a facultative anaerobic *Lactococci* spp. that grows poorly in both air and under anaerobic conditions. It usually exists in the reproductive tract and urine of animals and humans (Rasmussen, 2013), and is rarely reported in silage. The relative abundance of *Lactobacillus* in the LPLB treatment reached 11.97% on day 3 of ensiling, which was significantly higher than that of other treatments. These results indicate that *L. plantarum* and *L. brevis* had a synergistic effect in the early stage of paper mulberry silage fermentation. Research has shown that during inoculation with *L. plantarum*, its competitiveness was low in the early days of silage (7 days), and the abundance of *Lactobacillus* remained at a low level, which could not establish an advantage in the early stage (Chen et al., 2020). In addition, the relative abundance of *Lactobacillus* substantially increased in the LP (from 35.96% to 66.33%) and LB (from 40.45% to 70.89%) treatments after 30 days of fermentation, but in the LPLB treatment, it only increased from 51.55% to 54.57%. A probable explanation is that the synergistic effect of *L. plantarum* and *L. brevis* on days 3 and 30 of fermentation weakened or was converted into competitive effects with the prolonged ensiling. Generally, the microorganism composition during the early stage of ensiling is complex because the acidic environment has not formed yet. *Lactobacillus* spp. grows slowly in the early stage, but it still tends to exceed the number of *Lactococcus* spp. in the late stage of silage (Keshri et al., 2018). However, in this work, the highest abundance of *Aerococcus* in the control throughout the 60-day silage fermentation period, indicating that the activity of epiphytic *Lactobacillus* spp. was weaker than that of *Lactococcus* spp. in the naturally fermented paper mulberry silage. The characteristics of paper mulberry are similar to those of alfalfa, with high CP content and buffering capacity, resulting in the pH of paper mulberry silage decreasing slowly in this study. After fermentation for 30 days, the pH of each treatment remained above 4.8 and decreased to approximately 4.5 after 60 days of fermentation. However, the growth of *Enterobacter* can be inhibited only when the environmental pH is below 4.2 (McDonald et al., 1991). In our study, there was no markedly change in the abundance of *Enterobacter* during the whole fermentation process, indicating that the bacterial community had an insufficient transfer from undesirable microorganisms to lactic acid-producing bacteria. This is consistent with the results of Wang et al. (2019), who proposed that high protein content and buffering capacity can prevent the pH of alfalfa silage from decrease, thereby promoting *Enterobacter* growth during silage.

Generally speaking, *Lactobacillus*, *Wechsler*, *Lactococcus*, *Leuconostoc*, and *Streptococcus* are ideal LAB for increasing LA production and lowering pH value (Dong et al., 2020). Guan et al. (2018) reported that the concentration of LA was highly positively correlated with *Pediococcus* and *Lactobacilli.* In the present study, the LA and AA contents were positively correlated with *Lactobacillus* after 3 days of ensilage, and the pH value was negatively correlated with *Lactobacillus* after 3 days of ensilage. This is because *Lactobacillus* produces LA and AA by fermenting substrate during ensiling to reduce the pH value. There was a negative correlation between AA and *Aerococcus*, Probably attribute to the inhibitory effect of AA on *Aerococcus*. *Aerococcus* was positively correlated with the NH_3_-N content on day 60 of ensilage, and its abundance in the control was significantly higher than that in the other inoculated silages, which may explain the significantly higher NH_3_-N content in the control than in the other inoculated treatments. *Aerococcus* is rarely detected in silage; thus, further study is need to explore its role in the ensilage system.

## Conclusion

Inoculation of *L. plantarum* and/or *L. brevis* could consume the same WSC content to maximize the inhibition of *Aerococcus* growth at the early stage of fermentation and effectively reduced the NDF content at the end of fermentation. Inoculation of *L. plantarum* could effectively maintain the acidic environment at the end of the fermentation system by maintaining the abundance of *Lactobacillus*, maximizing the preservation of DM and crude protein, and reducing crude protein degradation. *L. brevis* inoculated alone or in combination with *L. plantarum* could significantly inhibit the growth of mold and improve the aerobic stability of paper mulberry silage. These results indicate that paper mulberry ensiling inoculation with *Lactiplantibacillus plantarum* and *Lactiplantibacillus brevis* could be a feasible way to improve silage quality.

## Data availability statement

The datasets presented in this study can be found in online repositories. The names of the repository/repositories and accession number(s) can be found in the article/supplementary material.

## Author contributions

YZ: designed the study and wrote the manuscript. RH: revised the manuscript and supervision. FZ and CM: formal analysis, project administration, and funding acquisition. HY and XW: validation and investigation. All authors contributed to the article and approved the submitted version.

## Funding

This work was financially supported by the Support Plan for Innovation and Development of Key Industries in South of Xinjiang (2022DB017) and China Agriculture Research System of MOF and MARA (CARS).

## Conflict of interest

The authors declare that the research was conducted in the absence of any commercial or financial relationships that could be construed as a potential conflict of interest.

## Publisher’s note

All claims expressed in this article are solely those of the authors and do not necessarily represent those of their affiliated organizations, or those of the publisher, the editors and the reviewers. Any product that may be evaluated in this article, or claim that may be made by its manufacturer, is not guaranteed or endorsed by the publisher.

## Footnotes

^1^ www.majorbio.com
